# Physiological, Genome-Wide Characterization and Expression Analysis of Aquaporin Gene Family of *Apocynum venetum* in Response to Abiotic Stress

**DOI:** 10.3390/genes17030352

**Published:** 2026-03-22

**Authors:** Wenhui Ma, Xiao Zhang, Yifan Huang, Yiling Liu, Wenlong Xie

**Affiliations:** 1 State Key Laboratory Incubation Base for Conservation and Utilization of Bio-Resource in Tarim Basin, College of Life Science and Technology, Tarim University, Alar 843300, China; whma04@foxmail.com (W.M.); tlmdxzx@163.com (X.Z.); yifan1207169783@foxmail.com (Y.H.); alexieviscera@foxmail.com (W.X.); 2 Xinjiang Production & Construction Corps Key Laboratory of Protected Agriculture, College of Horticulture and Forestry, Tarim University, Alar 843300, China

**Keywords:** *Apocynum venetum* L., aquaporin, genome-wide analysis, abiotic stress, gene expression, physiological indicators

## Abstract

**Background:** *Apocynum venetum* L., a saline–alkali-tolerant plant, is a valuable resource for forage, textile, and medicinal purposes. This study aimed to identify the AQP gene family in *A. venetum* genome-wide and explore their potential functions under abiotic stress. **Methods:** Gene identification, phylogenetic relationships, structural features, and evolutionary patterns were analyzed, along with gene expression patterns and correlations with physiological traits. **Results:** Phylogenetic analysis classified the 25 candidate AvAQP genes into five distinct subgroups, with members exhibiting conserved gene structures, motifs, and phosphorylation patterns. Subcellular localization predictions indicate targeting primarily to the plasma membrane or the vacuole, with one isoform (AvTIP5;1) predicted to localize to both. Synteny analysis revealed three intraspecific and multiple interspecific gene pairs (26 with *Arabidopsis thaliana* and 34 with *Medicago truncatula*). In silico promoter analysis identified 49 cis-regulatory elements associated with phytohormone response, stress signaling, and development, providing preliminary clues for their possible involvement in diverse biological processes. qPCR profiling under abiotic stress demonstrated tissue-specific expression patterns among AvAQP members under different stress conditions. Correlation analyses between gene expression and physiological indicators (growth- and water-related traits) were predominantly positive, with only a few negative correlations under stress conditions, suggesting that AvAQP expression may be associated with plant physiological status. **Conclusions:** This study presents a comprehensive analysis of the AQP family in *A. venetum* providing a foundation for further functional characterization of these genes in response to abiotic stress.

## 1. Introduction

*Apocynum venetum* L. is an erect perennial half-shrub belonging to the family Apocynum [1]. Distributed in Northern China between 35° N and 45° N, this species typically inhabits saline deserts, desert margins, riverbanks, and floodplains [2]. Its extensive root system contributes to soil stabilization and erosion control, playing an important role in the conservation of fragile Gobi Desert ecosystems [3]. Notably, moderate drought stress has been shown to enhance seed germination in *A. venetum* [4], suggesting adaptive strategies to water-limited environments. These ecological characteristics underscore the species’ resilience to arid conditions and highlight its potential as a model for studying plant adaptation to water deficit.

Despite its recognized ecological importance, the molecular mechanisms underlying *A. venetum*’s drought adaptation remain largely unexplored [5,6]. Water availability is a primary limiting factor in the desert habitats where *A. venetum* thrives, and plant survival under such conditions depends critically on efficient water transport and hydraulic regulation [7,8]. Aquaporins (AQPs) are membrane channel proteins that facilitate water movement across cellular membranes and play central roles in plant water relations, hydraulic conductivity, and responses to drought and salinity stress [9]. The classification system for AQPs in higher plants, according to structural and functional characteristics, encompasses five subfamilies: plasma membrane intrinsic proteins (PIPs), tonoplast intrinsic proteins (TIPs), nodulin26-like intrinsic proteins (NIPs), small basic intrinsic proteins (SIPs), and X-intrinsic proteins (XIPs) [10]. While PIPs, XIPs, SIPs, and a subset of NIPs localize to the plasma membrane, TIPs are predominantly tonoplast-resident. Notably, NIPs can also be present on the endoplasmic reticulum membrane [10].

Aquaporins (AQPs) are involved in many physiological and metabolic processes [11], and their activity is tuned by different environmental and developmental signals, including phytohormones and abiotic/biotic stresses [12]. Aquaporins maintain water homeostasis, enabling cell expansion, seed germination, and root growth while enhancing plant resilience to drought, salinity, and cold stress [13]. Other than active transport, AQPs play a role in signal transduction, ion homeostasis, and metabolism. For example, *AtPIP1;2* facilitates CO_2_ transport, *NIP2;1* plays a lactate channel during hypoxia, and *MaSIP2-1* overexpression enhanced drought and cold tolerance in banana [14,15,16].

In salt stress responses, AQPs enhance tolerance through precise regulation. Overexpression of *AcPIP2* and *AcNIP5;1* in Arabidopsis improved drought and salt tolerance [17], *MzPIP2;1* in *Malus zumi* and *SpAQP1* in *Sesuvium portulacastrum* were upregulated to enhance leaf growth [18,19], and barley *HvPIP1;6* was upregulated to promote leaf growth under salt stress [20]. Rice *OsPIP1-1* or *OsPIP2-2* overexpression in *Arabidopsis thaliana* enhanced drought resistance [21,22], and *PvPIP2;9* overexpression in *Panicum virgatum* increased plant height, biomass, and cellulose [23]. Rice *OsPIP2* overexpression improved survival [24], and transgenic banana lines overexpressing *MaSIP2-1* had better tolerance to drought and cold [16], and *TsTIP1;2* overexpression in Arabidopsis improved tolerance to drought, salt, and oxidative stress [25]. Note that low-temperature soil induces significant hydraulic resistance in sensitive plants, causing water imbalance, influencing AQP regulation: low temperature induces expression of TIP in tulip stems, and dephosphorylates plasma membrane aquaporins during petal closure [26,27,28]. In maize, water channel proteins restore root hydraulic conductance after cold injury [29]. These functions highlight the regulatory roles of AQPs in plant adaptation and provide targets for stress resistance breeding.

To date, most studies on aquaporin (AQP) gene families have focused on model plants and economically important crops, whereas desert species remain largely unexplored [30,31]. This is particularly true for *A. venetum*, a desert plant whose AQP repertoire has not yet been characterized at the genome-wide level, limiting our understanding of its adaptive strategies to extreme environments. The recent publication of the *A. venetum* genome [32] and subsequent improved assemblies [33] now provide a valuable foundation for such analyses. This study was designed with two primary aims. First, we aimed to establish the foundational genomic annotation of the AQP gene family in *A. venetum* through genome-wide identification and structural characterization. Second, and as the core objective, we sought to identify and prioritize stress-responsive AQP candidate genes. To achieve this prioritization, we analyzed their expression profiles under abiotic stress and, crucially, integrated these expression patterns with key physiological parameters. This combined analysis of expression and physiological traits was employed to highlight those AQP genes most strongly linked to the plant’s physiological adaptation under stress, thereby refining the candidate list for future functional validation. Collectively, this work provides the first comprehensive AQP resource for *A. venetum* and a prioritized set of candidates for elucidating their roles in abiotic adaptation.

## 2. Materials and Methods

### 2.1. Experimental Materials and Treatments

*A. venetum* seedlings were grown in vitro for three months, followed by three months of acclimation in a greenhouse under controlled conditions (25 °C, 16 h light/8 h dark photoperiod, light intensity: ~3000 lux). The plants were cultivated in a mixture of peat and vermiculite (1:1, *v*/*v*) as the growth substrate. Plants were placed in artificial climate chambers at 4 °C, 10 °C, 35 °C, and 40 °C for 24 h, with plants maintained at 25 °C serving as the control. In parallel, another set of seedlings was subjected to salinity stress (NaCl) and alkalinity stress (Na_2_CO_3_ and NaHCO_3_) for 24 h. The applied concentrations were 100 mmol/L and 300 mmol/L for both stress types, with water-treated plants used as the corresponding control. The pH ranged from 7.03 to 7.29 under salinity stress and from 9.84 to 10.69 under alkalinity stress. The experiment included five biological replicates. 

At the time of sampling, all plants were in the vegetative growth stage, each possessing 5–6 fully expanded leaves and showing no visible floral buds. This specific developmental stage (approximately 6-month-old plants with 5–6 fully expanded leaves) was selected as the treatment onset. For leaf samples, the third fully expanded leaf from the shoot apex (counted basipetally) was collected. For stem samples, the middle internode of the main stem was excised. For root samples, approximately 1 cm segments were taken from the primary root, positioned 1–2 cm behind the root tip.

For each biological replicate (one individual plant), tissues were collected as described above. Immediately after harvesting, each sample was divided into two portions. One portion was used immediately for the quantification of growth parameters (plant height, root length, fresh weight, dry weight, and tissue water content). The other portion was snap-frozen in liquid nitrogen and stored at −80 °C for subsequent RNA extraction and gene expression analysis. In this way, both physiological and molecular data were derived from the same set of biological replicates, enabling direct correlation analyses.

### 2.2. Identification, Physicochemical Analysis, and Classification of AvAQPs

The whole genome sequence of *A. venetum* was retrieved from NCBI. Protein sequences of the AQP families from *A. thaliana* and *Medicago truncatula*, together with the corresponding AQP hidden Markov model (HMM) profile, were obtained from Ensembl Plants and the Pfam database, respectively [34]. Using the HMMER tool (version 3.0), we performed seed alignment construction and a local hmmsearch. Candidate polypeptide sequences were subsequently extracted with SeqHunter (version 1.0), employing an E-value cutoff of 1 × 10^−10^. To eliminate false positives, the resulting candidates were subjected to multiple filtering steps. Specifically, each candidate was examined using the SMART tool (version 10.0) to verify the presence of a complete MIP family domain; sequences lacking this domain were discarded. Additionally, the presence of the two canonical NPA (Asn-Pro-Ala) motifs, characteristic of functional aquaporins, was assessed through multiple sequence alignment with reference AQP sequences. Candidates missing either or both NPA motifs were considered likely pseudogenes or misannotations and were excluded from subsequent analyses.

A multiple sequence alignment of the AQP proteins from *A. venetum*, *A. thaliana*, and *M. truncatula* was conducted using Clustal X (version 1.83) [35]. Finally, a phylogenetic tree was generated in MEGA X (version 11.0.13) employing the maximum likelihood method with 1000 bootstrap replicates. Additional phylogenetic trees of the AvAQP gene family were constructed using TBTools 2.400 under default parameters and visualized with the online iTOL tool (https://itol.embl.de/, accessed on 23 May 2024) [36].

The physicochemical properties of the screened AvAQP protein sequences were analyzed using the ExPASy ProtParam tool (https://web.expasy.org/protparam/, accessed on 23 May 2024). These included amino acid composition, molecular weight, theoretical isoelectric point (p*I*), instability index, aliphatic index, and grand average of hydropathicity (GRAVY). Transmembrane domains (TMDs) were predicted using the TMHMM server (version 2.0) (https://services.healthtech.dtu.dk/service.php? TMHMM-2.0, accessed on 23 May 2024). Subcellular localization was predicted using Plant-mPLoc. Potential phosphorylation sites (Ser, Thr, and Tyr) were identified using Scansite 4.0 (https://scansite4.mit.edu/#calcComposition;s=20f97e02-6a89-435f-9f4c-9d6190e7c2c0, accessed on 30 November 2025) [37].

### 2.3. Gene Structure, Conserved Motif, and Conserved Domain Analysis of AvAQPs

The gene structures of *A. venetum* AQP (AvAQP) family members were characterized using the online tool MEME (https://meme-suite.org/meme/tools/meme, accessed on 23 May 2024) with default parameters to identify conserved motifs [35]. Exon–intron organization was determined using the Gene Structure Display Server (GSDS 2.0, http://gsds.gao-lab.org/, accessed on 23 May 2024) based on alignments between genomic DNA and corresponding cDNA sequences. Additionally, conserved domains were confirmed using the batch CD-search tool from NCBI (https://www.ncbi.nlm.nih.gov/cdd/, accessed on 23 May 2024).

### 2.4. Promoter and Cis-Regulatory Element Analysis of AvAQPs

To identify cis-acting elements, the 2000 bp promoter region upstream of the start codon was analyzed using PlantCARE (http://bioinformatics.psb.ugent.be/webtools/plantcare/html/, accessed on 23 May 2024) [38]. The locations of predicted elements were then mapped using GSDS.

### 2.5. Chromosomal Distribution and Syntenic Analysis of AvAQPs

MCScanX (version 1.0.0) analysis was employed to identify tandem or segmental duplication events within the *A. venetum* AQP gene family. Furthermore, synteny analysis with *A. thaliana* and *M. truncatula* was performed using the One Step MCScanX module in TBtools to infer evolutionary relationships [39]. The generated synteny plots were exported in SVG format and visually enhanced using Adobe Illustrator software (version 16.0.0).

### 2.6. Analysis of the Gene Regulatory Network of the AvAQP Gene Family

To explore potential interactions among AvAQP proteins, the amino acid sequences were submitted to the STRING database (https://string-db.org/, accessed on 25 May 2024), and interactions were predicted based on homology to *A. thaliana* proteins. This approach infers putative functional associations by transferring known interaction data from orthologous AQP interactors in the well-annotated *A. thaliana* reference network [40]. This approach infers putative functional associations by transferring known interaction data from orthologous AQP interactors in the well-annotated *A. thaliana* reference network. It is important to note that this approach provides a predictive interaction framework based on orthology, rather than experimental evidence of physical interactions in *A. venetum*. The resulting interaction data were imported into Cytoscape (version 3.10.3) for visualization and network layout optimization. As such, the resulting network should be interpreted as an exploratory tool for hypothesis generation rather than a definitive map of AvAQP interactions.

### 2.7. Expression Patterns of AvAQP Genes Under Various Abiotic Stress Conditions

For gene expression analysis, a subset of treatment conditions was selected based on preliminary physiological screening and experimental capacity. These included three temperature treatments: 4 °C (extreme cold), 25 °C (control), and 40 °C (extreme heat), as well as salinity and alkalinity treatments using 0 and 300 mmol/L NaCl, NaHCO_3_, and Na_2_CO_3_, respectively. These endpoints were chosen to maximize contrast and detect robust expression changes associated with extreme stress conditions, while intermediate treatments (10 °C, 35 °C, and 100 mmol/L) were assessed only at the physiological level.

Total RNA was extracted using the FastPure Plant Total RNA Isolation Kit (TianGen, Beijing, China), and its quality was verified by electrophoresis on an RNase-free 1% agarose gel. Subsequently, cDNA was synthesized with the BeyoRT II First Strand cDNA Synthesis Kit (Beyotime, Shanghai, China). Quantitative real-time PCR (qRT-PCR) primers were designed based on the coding sequences (CDS) of AvAQP genes, with *A. venetum* β-actin serving as the internal reference (Table S1). Amplifications were performed on an Archimed R4 instrument (RocGene, Beijing, China) under the following program: 95 °C for 10 min, followed by 40 cycles of 95 °C for 15 s and 60 °C for 1 min. All primer melting curves displayed single sharp peaks, confirming specific amplification without non-specific products and demonstrating primer suitability for subsequent experiments (Figure S1). Each condition included five biological replicates, and gene expression levels were quantified using the 2^−ΔΔCT^ method [38]. Significant differences in gene expression between each stress treatment and its corresponding control group for a given tissue were determined by one-way analysis of variance (ANOVA) followed by Duncan’s multiple range test (*p* < 0.05). The statistical results of these comparisons are detailed in Supplementary Table S2.

### 2.8. Measurement of Growth and Water Physiology Indicators

We measured plant height and root length after stress treatments, then collected samples from roots, stems, and leaves with five biological replicates per treatment. Moisture content of plant tissues was calculated using Equation (1) [41]:

(1) $$M_{c}\left(\%\right)\;=\;\frac{m_{\mathrm{fresh}}-m_{\mathrm{dry}}}{m_{\mathrm{fresh}}}\times 100$$

where M_c_ represents the fresh weight water content of the leaves, m_fresh_ is the fresh weight of plant tissues, and m_dry_ is the mass after drying at 105 °C for 24 h.

### 2.9. Statistical Analysis

Statistical analyses were performed using SPSS software (version 26.0). Differences among treatment groups were assessed by one-way analysis of variance (ANOVA) followed by Duncan’s multiple range test, with statistical significance set at *p* < 0.05. Correlation analyses and data visualization were conducted using R (version 4.5.0). Specifically, the R packages *corrplot*, *psych*, *ggcorrplot*, *vcd*, and *ggplot2* were employed for statistical computations and graphical rendering. Expression heatmaps were generated using the *pheatmap* package in R to visualize the expression profiles of *AvAQP* genes across different tissues and stress treatments.

## 3. Results

### 3.1. Identification of AQP Gene Family in A. venetum

Based on the genome-wide data of *A. venetum*, a hidden Markov model corresponding to aquaporins (PF00230) was used as the query to identify candidate sequences. This search retrieved 25 polypeptides containing the conserved AQP motifs. The physicochemical properties of these AvAQP proteins were analyzed using the ExPASy-ProtParam tool (https://web.expasy.org/protparam/, accessed on 23 May 2024) (Table 1). The encoded proteins ranged from 241 to 331 amino acids in length, with molecular weights between 25.20 and 35.65 kDa and theoretical isoelectric points (p*I*) ranging from 5.07 to 9.43.

In silico subcellular localization predictions indicated that AvAQPs may localize to multiple compartments, such as the plasma membrane and vacuole. Transmembrane domain (TMD) predictions revealed that the majority (80%) of the AvAQP members conformed to the canonical six-TMD topology characteristic of most plant AQPs. However, three members were predicted to possess five TMDs, and two others were assigned only two TMDs. Given that such deviations from the typical AQP architecture are rare and may indicate potential issues in gene model prediction or incomplete annotation, we re-examined the corresponding genomic loci and coding sequences. In all cases, the two-TMD and five-TMD predictions could be attributed to either N- or C-terminal truncations in the gene models, or to low confidence in the secondary structure assignment for regions flanking the highly conserved TMD2 and TMD5. We therefore suggest that these members should be regarded as candidates for further experimental validation, rather than definitive AQP homologs. 

The aliphatic index ranged from 93.54 to 118.43, while the instability index varied from 25.92 to 42.25. Only one protein (AvNIP2;1) was classified as unstable, having an instability index greater than 40. The grand average of hydropathy (GRAVY) values for the AvAQP proteins ranged from 0.319 to 1.041. It is important to note that, according to the standard definition, a positive GRAVY value indicates a net hydrophobic character, whereas a negative value would suggest a predominance of hydrophilic residues. Therefore, the observed positive GRAVY values imply that most AvAQP members are, on average, slightly hydrophobic, which is consistent with the presence of transmembrane domains rich in hydrophobic amino acids. This finding does not contradict their known functions in membrane-associated water transport, as AQPs also contain highly polar loops and selectivity filter regions that facilitate water passage despite the overall hydrophobic nature of the TMDs.

Phosphorylation site prediction using Scansite4 identified between 33 and 67 putative sites per protein, composed of serine (Ser), threonine (Thr), and tyrosine (Tyr) residues. AvNIP2;1 had the highest number of Ser residues, while AvPIP1;1 had the fewest. AvNIP6;1 showed the most Thr residues, and AvTIP2;1 and AvTIP3;1 had the fewest. AvPIP2;2 contained the highest number of Tyr residues, and AvNIP6;1 the lowest.

### 3.2. Phylogenetic Analysis and Classification of AvAQP Genes

**Figure 1:**
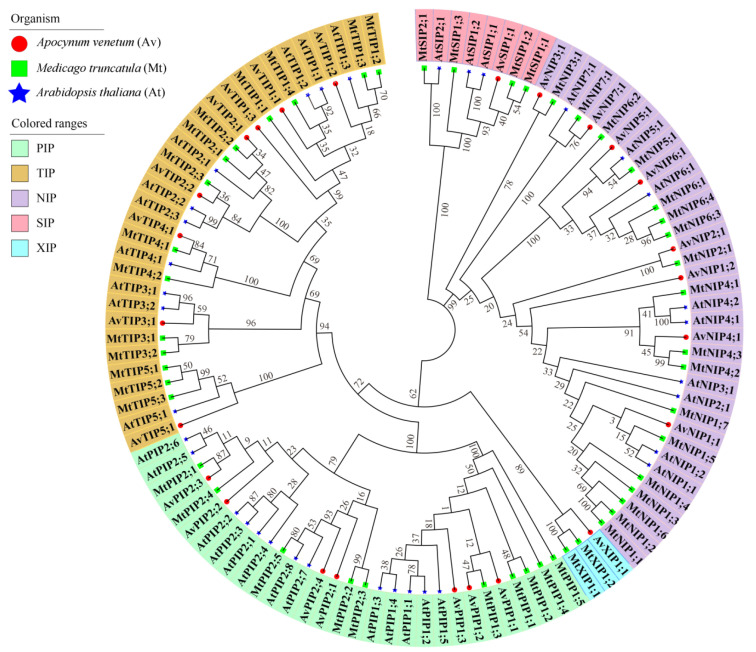
Phylogenetic analysis of AQP genes in *A. venetum*, *M. truncatula*, and *A. thaliana*. The AQP genes are denoted by red, blue, and green dots for *A. venetum*, *M. truncatula*, and *A. thaliana*, respectively. The subgroups are delineated by colored fan-shaped partitions homologous to five AQP in *A. venetum*.

This study constructed a phylogenetic tree based on the similarity of aquaporin (AQP) protein sequences in *A. venetum*, *A. thaliana*, and *M. truncatula* to elucidate the evolutionary relationships among AQP family genes and investigate their homology. The AQP proteins from *A. venetum* were clustered into five subfamilies: the TIP subfamily contained eight members, the PIP subfamily contained seven members, the NIP subfamily contained eight members, the SIP subfamily contained one member, and the XIP subfamily contained one member (Figure 1).

### 3.3. Gene Structure and Conserved Domain Analysis of AvAQPs

**Figure 2:**
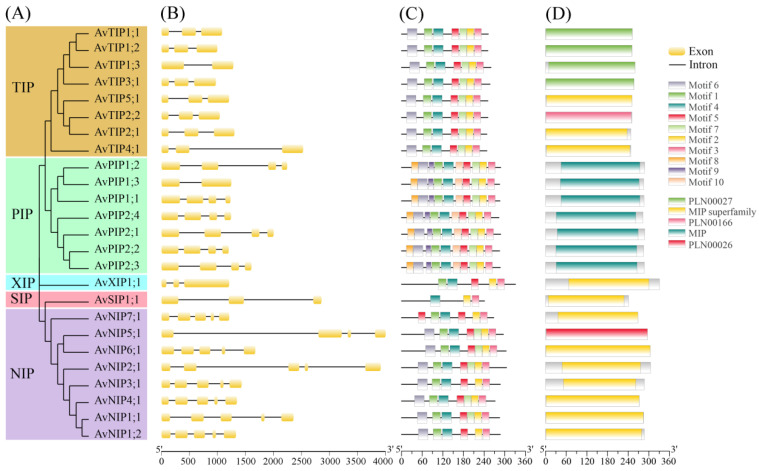
Phylogenetic relationships, gene structure, conserved motifs, and protein domains of AQP genes in *A. venetum*. (**A**) A maximum likelihood phylogenetic tree of AvAQP genes, constructed using MEGA-X; (**B**) exon–intron structure of AvAQP genes, analyzed using the GSDS tool; (**C**) motif composition of AvAQP proteins, visualized with TBtools; distinct colored boxes indicate motifs comprising specific amino acid sequences; (**D**) domain architecture of AvAQP proteins, visualized with TBtools; different colored boxes represent conserved domains.

Gene structure analysis revealed that members of each aquaporin subfamily generally shared a conserved exon–intron organization (Figure 2B). Specifically, in the TIP subfamily, all members except AvTIP1;3 (two exons and one intron) contained three exons and two introns. Similarly, all members of the PIP subfamily, with the exception of AvPIP1;3 (which has two exons and one intron), possess four exons and three introns. Within the NIP subfamily, AvNIP5;1 was an exception with four exons and three introns, while the remaining members had five exons and four introns. Both the SIP and XIP subfamilies consisted exclusively of three exons. Moreover, phylogenetic closeness within each subfamily correlated with similarity in exon number and arrangement, indicating that gene structure is largely conserved among evolutionarily related proteins.

MEME analysis identified ten conserved motifs across the AvAQP protein family (Figure 2C). The overall motif architecture was highly conserved, with motifs 2, 3, and 4 shared by most subfamilies (PIPs, TIPs, NIPs, SIP, and XIP). Members within each subfamily generally shared similar motif types and copy numbers. Notably, the PIP subfamily contained distinctive motifs 8, 9, and 10, which were absent in other subfamilies. The motif organization of the TIP and NIP subfamilies was largely similar, except in *AvNIP1;2*, *AvNIP3;1*, and *AvNIP7;1*. Overall, motif type and arrangement were highly conserved within each subfamily, while certain subfamily-specific motifs likely reflect functional conservation among related aquaporins. Domain analysis indicated that all AvAQP proteins contained the characteristic MIP superfamily domain, confirming their conserved functional role (Figure 2D).

### 3.4. Cis-Regulatory Element Analysis of AvAQPs

**Figure 3:**
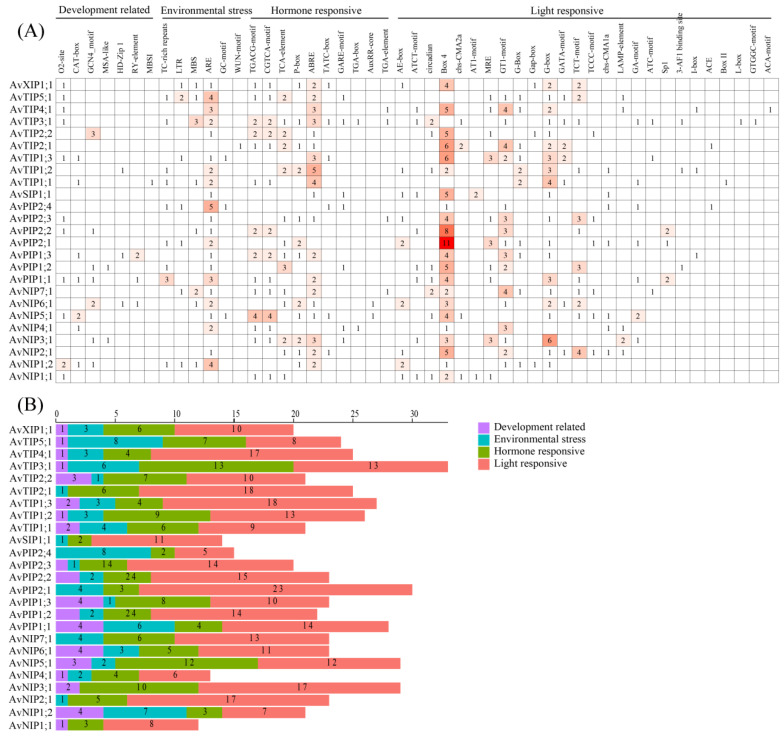
Prediction of cis-regulatory elements in the promoter regions of AvAQP genes. (**A**) The putative cis-elements were quantified and functionally classified based on their established roles in gene transcriptional regulation; darker colors correspond to higher abundances of the respective cis-regulatory elements. (**B**) distribution diagram of cis-elements for AvAQP gene promoters, with diverse colored geometric figures representing distinct cis-elements.

To explore the potential transcriptional regulatory mechanisms of the *A. venetum* AQP gene family, we predicted cis-acting elements within the 2000 bp promoter region upstream of each gene. The results for 25 representative AQP genes are shown in Figure 3. Based on their functions, cis-acting elements were categorized into four groups: development-responsive elements, environment-responsive elements, hormone-responsive elements, and light-responsive elements (Figure 3A).

Promoter analysis revealed the presence of development-related elements, including those associated with flavonoid biosynthesis, meristem expression, endosperm expression, and circadian rhythm. Environment-responsive elements included motifs related to anaerobic induction, drought induction, hypoxia stress, low-temperature response, and defense response. Hormone-responsive elements for auxin, abscisic acid, ethylene, gibberellin, methyl jasmonate, and salicylic acid were also detected. Light-responsive elements were widely distributed, with some AvAQP genes containing multiple photoperiod-related motifs (e.g., Box 4 and G-box) in their promoters (Figure 3B).

It is important to note that the identification of these elements in promoter regions only provides a basis for hypothesizing about possible regulatory functions. The presence of a given cis-element does not, by itself, establish that the corresponding gene is regulated by the associated pathway, nor does it directly demonstrate photoperiod involvement, oxidative-stress specialization, or epigenetic modification. For example, although the promoter of AvPIP2;1 contains 11 photoperiod-related elements, this observation can only be taken as an indication of potential responsiveness to light signals, pending experimental validation. Similarly, the detection of hypoxia-responsive elements in TIP, PIP, and NIP subfamilies may point to a possible involvement in oxygen-related signaling, but conclusions about oxidative stress specialization or epigenetic regulation require independent biochemical or genetic evidence [42].

Thus, while the widespread occurrence of ABA-responsive elements suggests that many AvAQP genes could integrate stress signals, any specific functional claims should be tempered until supported by direct experimental data.

### 3.5. Genome Distribution and Syntenic Analysis of AvAQP Genes

**Figure 4:**
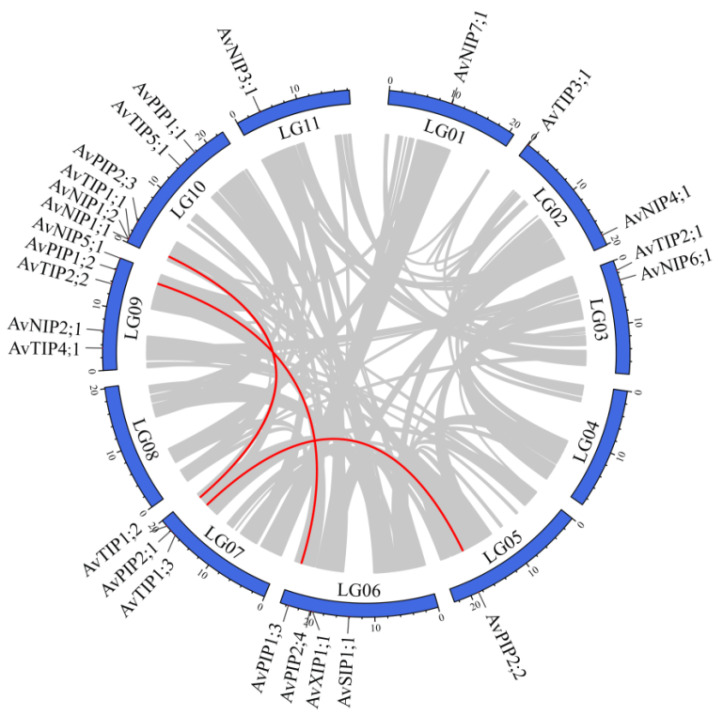
Chromosomal localization and intraspecific synteny of AvAQP genes. The blue areas indicate chromosomes, with the scale bar representing megabases (Mb). The gray areas indicate collinear regions, and the red lines indicate syntenic relationships between AQP genes of *A. venetum*.

The chromosomal localizations and genome-wide duplication events of the AvAQP genes were mapped using TBtools (Figure 4). The genome of *A. venetum* comprises 11 chromosomes. The 25 candidate AvAQP genes are distributed across all chromosomes except Chr4 and Chr8, indicating a broad genomic distribution. Intraspecific collinearity analysis identified three pairs of segmentally duplicated genes exhibiting genomic synteny within the AvAQP family. This duplication has likely driven the proliferation of AvAQP homologs across the genome, thereby increasing the potential for evolutionary diversification.

**Figure 5:**
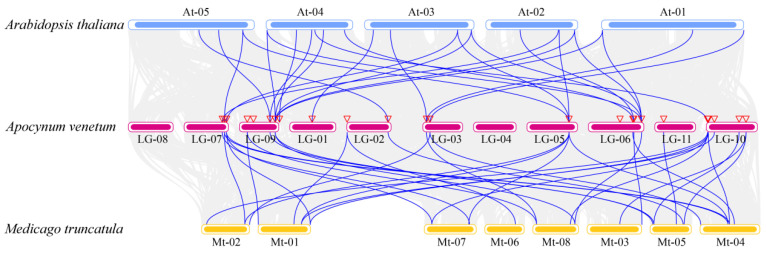
Interspecific synteny of AQP genes across different species. The blue, pink, and yellow round rectangles indicate chromosomes of *A. thaliana*, *A. venetum*, and *M. truncatula*, respectively. The gray areas indicate collinear regions, and the blue lines indicate syntenic relationships between AQP genes across different species. The triangles indicate the chromosomal locations of the AvAQP genes.

To further investigate the phylogenetic mechanisms of the AvAQP gene family, we constructed a genome-wide collinearity map for *A. thaliana* (At), *M. truncatula* (Mt), and *A. venetum* (Av), with a focus on AQP gene family members that showed collinear relationships across these species. The analysis identified 26 syntenic gene pairs involving 14 AvAQP genes and 20 AtAQP genes, as well as 34 syntenic gene pairs between 21 AvAQP genes and 25 MtAQP genes (Figure 5). These results suggest a high degree of homology among the AQP families of *A. venetum*, *A. thaliana*, and *M. truncatula*. The syntenic gene pairs identified are likely to share similar functional properties and may have descended from a common ancestor.

### 3.6. Analysis of the Protein Interaction Network of the AvAQP Gene Family

**Figure 6:**
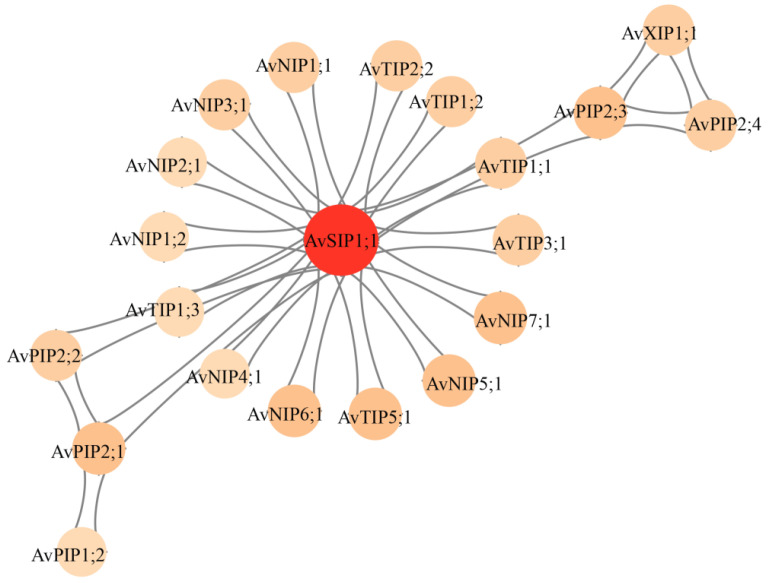
The protein–protein interaction network of AvAQPs was constructed using the STRING database. Nodes represent AvAQP proteins and their interacting partners, and edges represent the associations between them, including known, predicted, and other interactions. The color of a node represents the number of edges, with darker colors indicating a higher number of connections.

To investigate the function of the AvAQP gene family, 25 AvAQP protein sequences were analyzed using the STRING online database. The resulting protein–protein interaction (PPI) network (Figure 6) contains 21 nodes and 156 edges, representing the proteins and their interactions, respectively. In this network, 21 of the 25 AvAQP members were found to have at least one interaction partner, and AvSIP1;1 displayed the highest number of connections, interacting with 17 other AvAQP proteins. This result indicates that, in the predicted interaction network, the ortholog of AvSIP1;1 in the reference species occupies a central position.

### 3.7. Expression Patterns of AvAQP Genes Under Abiotic Stress

**Figure 7:**
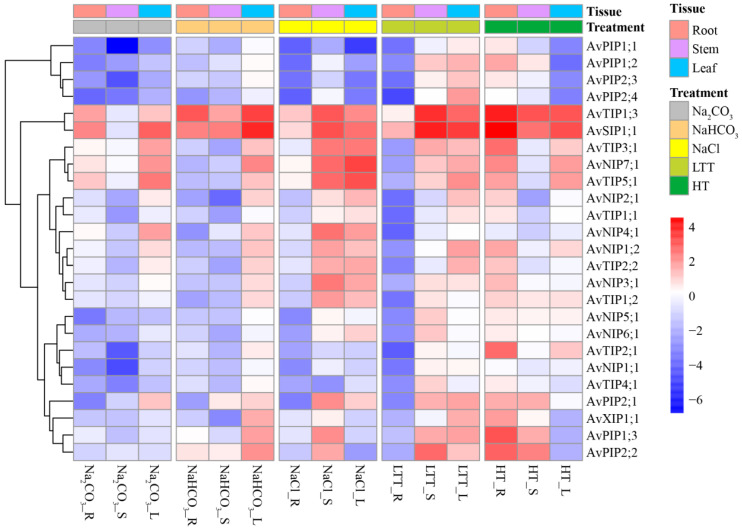
Expression heatmap of the *A. venetum* AQP gene family under abiotic stress. LTT, low-temperature treatment; HT, high-temperature treatment.

We analyzed the expression patterns of 25 AvAQP gene family members under five abiotic stress conditions and examined their tissue-specific expression. The expression changes depicted in the heatmap (Figure 7) are based on statistically significant differences (*p* < 0.05, ANOVA with Duncan’s test) between stress treatments and their respective controls, as detailed in Supplementary Table S2. Figure 7 shows that the 25 AvAQP genes responded to Na_2_CO_3_, NaHCO_3_, NaCl, low-temperature (LT), and high-temperature (HT) treatments to varying degrees.

Na_2_CO_3_ stress caused tissue-specific expression changes. In roots, *AvPIP1;1* was significantly downregulated, while other genes remained largely unaffected. Stems showed broad downregulation with only minor increases in a few genes. Leaf expression profiles were mostly stable, with the exception of a slight decrease in *AvPIP1;1*. *AvSIP1;1* was markedly upregulated across all tissues under NaHCO_3_ stress, identifying it as the central responder. *AvPIP2;1* showed a weak increase in roots and stems, while other genes were generally slightly downregulated. NaCl stress triggered significant upregulation of *AvSIP1;1*, *AvTIP3;1*, and *AvNIP7;1* in roots and stems, with only *AvPIP1;1* being weakly downregulated. NaCl stress upregulated more genes than Na_2_CO_3_ and NaHCO_3_ treatments (Table S2). Low-temperature and high-temperature stresses consistently upregulated *AvSIP1;1* and *AvTIP1;3* in all tissues. However, low-temperature induced a broader response, clearly upregulating *AvNIP7;1* and *AvPIP2;1* in roots and stems, and more genes in leaves than high-temperature stress.

### 3.8. Correlation Analysis of Physiological Indicators Under Abiotic Stress

**Figure 8:**
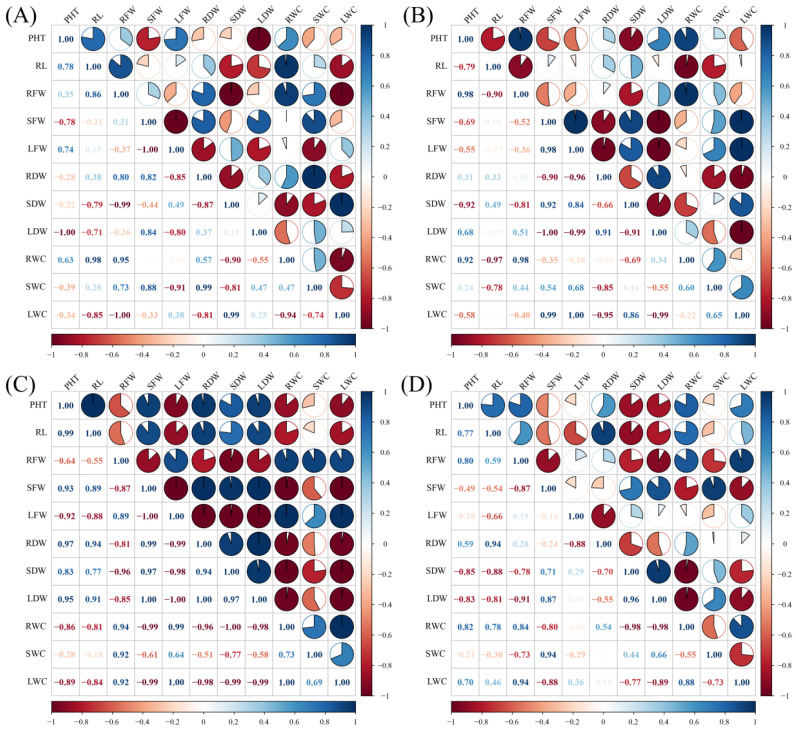
Correlation heatmaps of physiological indices under various stress conditions. (**A**) Na_2_CO_3_ stress; (**B**) NaHCO_3_ stress; (**C**) NaCl stress; (**D**) Combined high and low temperature stress. Abbreviations: PHT, plant height; RL, root length; RFW, root fresh weight; SFW, stem fresh weight; LFW, leaf fresh weight; RDW, root dry weight; SDW, stem dry weight; LDW, leaf dry weight; RWC, root water content; SWC, stem water content; LWC, leaf water content. The color gradient from blue to red represents correlation coefficients ranging from −1 (strong negative correlation) to +1 (strong positive correlation).

Correlation analyses among physiological indices under the four abiotic stress conditions are summarized in Table S3 and Figure 8.

Under Na_2_CO_3_ stress (Figure 8A), plant height (PHT) and root fresh weight (RFW) exhibited a positive correlation, while both were negatively correlated with stem fresh weight (SFW). Leaf fresh weight (LFW) was positively correlated with SFW but negatively correlated with root dry weight (RDW). Among dry weight components, stem dry weight (SDW) and leaf dry weight (LDW) were negatively correlated. All tissue water content parameters (SWC, RWC, LWC) were positively correlated, a pattern distinct from that observed under NaCl stress. Root water content (RWC) was negatively correlated with root length (RL) but positively correlated with RFW.

Under NaHCO_3_ stress (Figure 8B), PHT and RL showed a strong positive correlation, while SFW and LFW were strongly negatively correlated. Dry weight components (RDW, SDW, LDW) were strongly positively correlated. RWC was positively correlated with SWC and LWC, but showed opposite associations with SFW (positive) and LFW (negative); all dry weight components correlated negatively with RWC.

Under NaCl stress (Figure 8C), RL, RFW, and RWC formed a cluster of positive correlations. In contrast, SFW and LFW were negatively correlated. Moisture indicators displayed mixed relationships, with RWC and SWC both negatively correlated with LWC.

Under combined high and low temperature stress (Figure 8D), dry weight indicators were strongly positively correlated, whereas fresh weight indicators were predominantly negatively correlated (e.g., between SFW and RFW). Moisture indicators showed complex interrelations: RWC was positively correlated with LWC, but both were negatively correlated with stem water content. Fresh weight and moisture indicators diverged, with RFW positively correlated with RWC and LWC, while SFW showed the opposite trend.

It is important to emphasize that these correlation patterns reflect associations observed over a short 24 h stress period and do not imply causal relationships among resource allocation, water-use efficiency, or AQP-mediated regulation. Further experimental investigation would be required to establish any mechanistic links.

## 4. Discussion

### 4.1. Genome-Wide Identification and Characterization of the AvAQP Gene Family

Water is essential for plant survival, with water molecules crossing cell membranes from the external environment into cells to participate in various physiological processes [26]. AQPs play a critical regulatory role in plant growth and development [11]. As an important class of membrane proteins, AQPs are involved in multiple functions, including water transport, cellular osmotic regulation, seed germination, lateral root formation, carbon fixation, nutrient uptake, and stress responses [43]. In this study, 25 AvAQP family members were identified from the *A. venetum* genome. Although AQPs are widely distributed in plant genomes and have been extensively characterized in many higher plant species, such as *Selaginella moellendorffii*, *Physcomitrella patens*, and maize [44,45,46], functional studies of the AQP gene family in *A. venetum* have not been reported to date. Given that AQPs are consistently associated with environmental stress tolerance, a comprehensive genome-wide analysis of this family in *A. venetum* is of considerable importance. These considerations further underscore the biological and agronomic significance of AQP genes in plants.

Phylogenetic analysis classified all AvAQPs into five subfamilies. Conserved domain analysis identified a characteristic core region within the AQP family, primarily composed of the MIP domain. Members within the same branch shared similar motif compositions, suggesting a closer evolutionary relationship [47]. Sequence analysis revealed that nearly all members contain Motif 2, Motif 3, and Motif 4. The high conservation of these motifs implies they may be functionally important, although their precise roles in *A. venetum* remain to be experimentally validated.

Cis-acting element analysis of the AvAQP gene promoter regions identified several elements associated with environmental stress and hormone responsiveness, including Box 4, G-box, ABRE, and ARE [48,49,50,51]. The presence of these elements suggests that the corresponding genes may be responsive to hormonal signals and abiotic stress, but such predictions require further experimental confirmation.

Analysis of chromosomal distribution revealed a non-random pattern of AvAQP genes across the 11 linkage groups (LG). LG09 exhibited the highest gene density, while no AQP family genes were detected on LG04. Some subfamily members appeared to cluster on specific LGs, which could be indicative of segmental duplication events, though this hypothesis needs additional support [52].

Synteny analysis between *A. venetum* and the model species *A. thaliana* and *M. truncatula* identified a number of orthologous AQP gene pairs [53]. The high degree of synteny suggests that these genes may have retained similar functions during evolution, but this remains a working hypothesis pending functional evidence.

In summary, the integration of phylogenetic, conserved motif, cis-element, synteny, and selection analyses provides a framework for generating hypotheses about the potential functions of AvAQP genes, particularly in relation to abiotic stress responses. These hypotheses warrant further experimental investigation.

### 4.2. Stress- and Tissue-Specific Expression of AvAQP Genes and Their Correlations with Physiological Adaptation

In contrast to well-studied model plants such as rice, tobacco, and Arabidopsis, the regulatory role of AQPs in desert plants remains poorly characterized [52,54,55]. Furthermore, how interspecies variation influences whole-plant AQP function under environmental stress has received limited attention [56]. To cope with abiotic stress, plants modulate the expression of distinct aquaporin genes to maintain cellular water balance and ion homeostasis. Under salinity and alkalinity stress, the upregulation of certain AQPs may potentially facilitate the removal of excess Na^+^ and help sustain cellular osmotic equilibrium [57]. During temperature stress, induced aquaporin expression is likely involved in regulating membrane fluidity and protecting intracellular structures [16].

Our data show that AvSIP1;1 was consistently upregulated in roots, stems, and leaves under all stress treatments. This pattern suggests a hypothesis that AvSIP1;1 may function as a broadly responsive gene in abiotic stress, but this role should be regarded as a working hypothesis. AvTIP3;1 and AvNIP7;1 also showed increased expression under NaCl, low-temperature, and high-temperature treatments, implying a possible role in managing ionic and thermal stress. Conversely, AvPIP1;1 was mostly downregulated or unchanged, which could indicate a less prominent or suppressive role in the stress-response network, although this contrasts with some previous reports [58,59].

Tissue-specific expression analysis revealed that roots were generally the most stress-sensitive, with the highest expression levels for many upregulated genes. Leaves showed the strongest transcriptional response to high-temperature stress, while stems displayed an intermediate pattern. These observations are consistent with a model in which different tissues may employ distinct subsets of AQPs to manage stress, but the model requires experimental validation.

Correlation analysis among physiological indicators revealed both positive and negative associations. Growth parameters showed strong internal consistency, and different stress treatments altered the strength and direction of these correlations. For example, under Na_2_CO_3_ and NaCl stress, some growth-related correlations approached 1.00, which may reflect a prioritization of biomass accumulation. Under NaHCO_3_ or temperature stress, the correlation between hydraulic and growth indicators decreased, potentially indicating reduced water-use efficiency. Negative correlations could suggest resource trade-offs, such as reduced leaf growth in favor of root and stem development, but these interpretations are speculative and based on correlative evidence.

The interplay between AQP expression and physiological adaptation is complex. In root tissues, upregulation of PIP family genes might potentially enhance water absorption and influence root length and fresh weight, which in turn could affect whole-plant biomass accumulation. TIP family genes may help maintain cellular turgor and stabilize water content during stress. The possible downregulation of leaf NIP genes under stress might correlate with reduced transpirational water loss, possibly redistributing water to other organs. However, these proposed mechanisms are based on correlative data and should be treated as testable hypotheses.

In summary, the data support a model in which aquaporin expression, water transport, and physiological adaptation are coordinately regulated under stress. Nevertheless, the specific functional roles of individual AvAQP members, and the causal relationships implied by the correlations, remain to be established experimentally.

Previous research demonstrated that in the roots of *Cucumis melo* L., high temperature and salinity treatments significantly downregulated the expression of *SIP1;1* [58]. In contrast, *AvSIP1;1* was consistently and significantly upregulated in *A. venetum* roots, stems, and leaves across all stress treatments, confirming its role as a core gene in general abiotic stress responses, as shown in Figure 6. In addition, the expression of *AvTIP3;1* and *AvNIP7;1* significantly increased in response to NaCl, low-temperature, and high-temperature treatments, suggesting their joint involvement in mitigating both ionic and thermal stresses in *A. venetum*. Conversely, the expression of *AvPIP1;1* was predominantly downregulated or unchanged under most conditions, indicating its possible role as a negative regulator within stress-response pathways. This finding is contrary to the results reported in previous studies [58,59]. Roots were identified as the most stress-sensitive tissue [56], exhibiting the highest expression levels for the majority of upregulated genes in *A. venetum*. In *A. venetum*, the leaves exhibited the most pronounced transcriptional response to high-temperature stress, containing the highest number of stress-induced genes. In contrast, the stem showed an intermediate level of responsiveness, with its expression patterns closely resembling those of the roots. In conclusion, this study illustrates that *A. venetum* modulates the expression of a range of aquaporin genes in a manner that is specific to both stress conditions and tissue types, thereby maintaining cellular homeostasis under abiotic stress.

The physiological indicators measured in this study exhibited mainly positive correlations, with a few negative associations. Growth parameters showed strong internal consistency, with plant height (PHT) closely associated with root length (RL), and significant intercorrelations among the fresh weights of root, stem, and leaf. Dry weight traits displayed even tighter synchrony, suggesting coordinated above- and below-ground biomass accumulation [60].

Different stress treatments appeared to modify the coordination among physiological traits, as indicated by changes in correlation patterns [60]. Under Na_2_CO_3_ and NaCl stress, correlations among growth-related indicators tended to increase, sometimes approaching 1.00, which may reflect a more tightly coupled growth pattern under these conditions. In contrast, NaHCO_3_ or temperature stress weakened the correlation between hydraulic indicators (e.g., shoot water content, SWC) and growth traits such as PHT (from 0.85 to 0.6). As water-use efficiency was not directly quantified, this change is interpreted here only as an alteration in the relationship between water status and growth, rather than an indicator of efficiency loss. Observed negative correlations could suggest shifts in resource allocation, although confirming true resource trade-offs would require biomass partitioning or isotopic analyses.

Aquaporin gene expression is hypothesized to influence water transport and related physiology, though direct measurements of ion fluxes, hydraulic conductance, stomatal behavior, membrane properties, or protein activity were not conducted here. Root-expressed PIP family genes are known from the literature to facilitate water uptake by increasing plasma membrane permeability [11,12]; their upregulation in our data could therefore contribute to root length (RL) and root fresh weight (RFW), with potential downstream effects on whole-plant growth as reflected in the RDW–SDW relationship. TIP family genes have been implicated in cellular turgor maintenance [56] and could help stabilize relative water content (RWC) and leaf water content (LWC) via tonoplast transport. Under saline conditions, their induction might assist osmotic adjustment, thereby reducing dehydration risk [27]. Leaf-expressed NIP genes were generally downregulated under stress; this pattern could be consistent with reduced transpirational water loss via limitation of apoplastic outflow, possibly favoring water redistribution toward roots and stems [58]. These interpretations are tentative and serve as working hypotheses for future testing.

It is important to note that our findings derive from a single *A. venetum* accession exposed to short-term stress in controlled growth chambers. Aquaporin regulation is known to be highly genotype-dependent, varying with genetic background, promoter architecture, and post-translational modification. Therefore, the candidate AQP responses observed here may not directly translate to other Apocynum genotypes, cultivated varieties, or even other saline/medicinal species. Field validation and comparative studies across accessions and species will be essential to determine the broader applicability of these results.

In terms of translational relevance, the identification of these candidate AQP genes offers several concrete opportunities. In breeding programs, germplasm screening for high root-specific PIP and TIP expression under saline or alkaline conditions could provide molecular markers for improved water uptake and turgor maintenance [61]. Rapid screening protocols using qRT-PCR or promoter-GUS fusions could preselect promising accessions before field trials. In stress management, knowledge of AQP expression patterns could guide irrigation scheduling or the choice of soil amendments, especially in marginal areas with poor water quality [62]. For production systems, deploying AQP-proficient genotypes may enable stable yields of this medicinally and economically valuable species under suboptimal conditions, expanding the range of cultivable land.

Taken together, our results outline a testable model linking aquaporin expression patterns with observed physiological adjustments under stress. The apparent coordination between root PIP/TIP expression and growth metrics, alongside the negative association of leaf NIP expression with certain water-related traits, may constitute a core component of *A. venetum*’s stress-responsive strategy. Further work measuring hydraulic conductance, stomatal aperture, and ion fluxes will be needed to validate these proposed links.

## 5. Conclusions

This study presents the first genome-wide characterization of the aquaporin (AQP) gene family in *A. venetum*, identifying a total of 25 AvAQP genes and phylogenetically classifying them into five subfamilies: seven PIPs, eight TIPs, eight NIPs, one SIP, and one XIP. Conserved motif analysis revealed evolutionary conservation within the PIP, TIP, and NIP subfamilies. We documented the expression profiles of these genes under multiple abiotic stress conditions, including Na_2_CO_3_, NaHCO_3_, NaCl, and temperature stress. Several AvAQP genes, notably *AvSIP1;1* and *AvTIP1;3*, showed responsiveness to multiple treatments, with AvSIP1;1 highlighted as a candidate stress-related member through protein interaction network analysis. Correlation analyses between AvAQP expression and physiological traits suggested possible involvement in water transport regulation, although these associations are considered preliminary.

By establishing the first comprehensive AQP dataset for *A. venetum*, this work provides a critical foundation for future functional studies, including gene validation and exploration of the molecular mechanisms underlying saline-alkali and temperature tolerance in this medicinally and economically important species.

## Figures and Tables

**Table 1:** Physicochemical properties and subcellular localization of *AvAQP* genes.

Subfamily	Gene	Amino Acid (AA)	Molecular Weight (kDa)	Isoelectric Point (p*I*)	Subcellular localization	TMD	Instability Index	Aliphatic Index	GRAVY	Phosphorylation Site	
										Ser/Thr/Tyr	Total
PIP	AvPIP1;1	286	30.83	8.96	Cyt	5	32.99	93.92	0.327	9/19/11	39
	AvPIP1;2	288	31.02	8.97	Cyt	6	28.06	95.94	0.319	14/16/10	40
	AvPIP1;3	285	30.71	8.88	Cyt	6	27.35	97.58	0.356	14/16/8	38
	AvPIP2;1	288	31.01	6.43	Cyt	6	35.23	93.54	0.465	12/18/12	42
	AvPIP2;2	285	30.49	7.61	Cyt	6	30.85	99.02	0.469	14/16/14	44
	AvPIP2;3	287	30.73	7.11	Cyt	6	35.17	100.98	0.495	16/15/11	42
	AvPIP2;4	283	30.36	8.82	Cyt	6	28.73	102.76	0.462	13/14/11	38
TIP	AvTIP1;1	252	25.76	5.14	Vac	6	25.92	113.49	0.84	18/13/6	37
	AvTIP1;2	251	25.83	5.35	Vac	6	28.77	109.72	0.841	19/16/6	41
	AvTIP1;3	260	27.06	6.64	Vac	6	27.09	116.31	0.873	16/15/6	37
	AvTIP2;1	248	25.20	5.76	Vac	7	28.34	118.43	1.041	17/9/7	33
	AvTIP2;2	251	25.44	5.07	Vac	6	29.24	115.54	0.944	22/11/9	42
	AvTIP3;1	257	27.17	8.08	Vac	6	35.38	101.48	0.571	12/9/9	30
	AvTIP4;1	248	25.97	6.02	Vac	6	27.44	116.85	0.831	13/13/7	33
	AvTIP5;1	251	26.07	7.9	Cyt, Vac	6	39.21	101.92	0.59	28/15/9	52
NIP	AvNIP1;1	285	30.23	9.43	Cyt	6	31.92	100.95	0.438	25/20/7	51
	AvNIP1;2	287	31.05	9.1	Cyt	5	33.64	97.28	0.327	27/16/8	51
	AvNIP2;1	305	32.61	8.33	Cyt	6	42.25	114.81	0.578	34/24/9	67
	AvNIP3;1	287	30.56	8.43	Cyt	6	34	102.1	0.601	23/20/8	51
	AvNIP4;1	287	31.05	9.1	Cyt	7	35.07	111.78	0.499	23/19/6	48
	AvNIP5;1	296	30.67	8.3	Cyt	6	31.72	99.63	0.488	24/22/6	54
	AvNIP6;1	304	31.28	8.67	Cyt	6	31.12	102.17	0.503	17/25/4	46
	AvNIP7;1	268	28.09	7.67	Cyt	6	39.43	116.12	0.653	31/14/8	53
SIP	AvSIP1;1	241	25.53	9.1	Cyt	5	28.92	110.71	0.837	15/17/10	42
XIP	AvXIP1;1	331	35.65	7.71	Cyt	6	39.28	110.79	0.566	26/16/7	49

Note: Abbreviations stand for the following: Cyt, cytomembrane; Vac, vacuole; Ser, serine; Thr, threonine; Tyr, tyrosine.

## Data Availability

Data are contained within the article. The original data presented in the study are openly available in the National Center for Biotechnology Information (NCBI) at PRJNA476646.

## Supplementary Materials

The following supporting information can be downloaded at https://www.mdpi.com/article/10.3390/genes17030352/s1, Table S1: qRT-PCR primer details for aquaporin genes from *A. venetum*; Table S2: ∆CT Values and ANOVA results from quantitative real-time PCR across different tissues and gene groups; Table S3: Comparative physiological profiling of *A. venetum* under various stress conditions; Figure S1: Melting curve analysis of 25 aquaporin genes from *A. venetum*.
