# Supplementary material for: Physiological, Genome-Wide Characterization and Expression Analysis of Aquaporin Gene Family of Apocynum venetum in Response to Abiotic Stress
Source: Genes (Basel). 2026 Mar 22;17(3):352. doi: 10.3390/genes17030352 (PMC13025683; doi:10.3390/genes17030352)
Supplement: Supplementary file 1 [file genes-17-00352-s001.zip › genes-4213071-supplementary.pdf]

Table S1. qRT-PCR primer details for aquaporin genes from *A. venetum*

Primer name	Primer sequence(5'→3')	Length (bp)	Efficiency (%)	R^{-2}
AvSIP1;1-F	GGGTCCTCGTAGTCCACTTAT	726	0.983	0.9991
AvSIP1;1-R	TCATGGAGGGTCCAGTGTAA			
AvXIP1;1-F	CTGCAGGGTTAGTTGGTCTTAT	996	0.964	0.9933
AvXIP1;1-R	ACGGCTTTGAGTGCTAGTG			
AvNIP1;1-F	TGTTTGTCATCTCCGGTGTC	858	0.965	0.9984
AvNIP1;1-R	CCCGGCAAACATCACATTTAG			
AvNIP1;2-F	CAACTCCTTGGCTCAACACTA	864	0.973	0.9978
AvNIP1;2-R	GATTGCATGTAGGAGCCTGAT			
AvNIP2;1-F	GTTGGAGCATGGGCCTATAA	918	0.955	0.9984
AvNIP2;1-R	CTCCTCGTGGAACAGAGTTT			
AvNIP3;1-F	GTCTGGTGGTGATGGCTTTA	864	1.041	0.9921
AvNIP3;1-R	CGTGCAGCAAAGGAAATCTG			
AvNIP4;1-F	TCTAGCAAGCGGCACATTAG	819	0.972	0.9958
AvNIP4;1-R	CCGGAGACGACAAACATAAGAA			
AvNIP5;1-F	CACACGTGTCTCACCGATTT	891	0.959	0.9916
AvNIP5;1-R	GGCCGTAGCACCAAATATCA			
AvNIP6;1-F	GGAGGAGTTACAGTTCCATCAG	915	0.967	0.9956
AvNIP6;1-R	GCGGTGACAACAAACATGAG			
AvNIP7;1-F	GATCATTAGGGCCAGCACTT	807	0.983	0.9999
AvNIP7;1-R	TACAAGAGCCCACCAGAAATG			
AvPIP1;1-F	TGGAGCAGCCATCATCTATAAC	861	1.02	0.9917
AvPIP1;1-R	GGATTGCCCTGATCACTACTT			
AvPIP1;2-F	CTCTCTATGACCAGGGCTATCT	867	0.958	0.9929
AvPIP1;2-R	CCACCATTGAGTCTCTCGTATG			
AvPIP1;3-F	CGCCACTTTCTTGTTCCTTTAC	858	0.968	0.9997
AvPIP1;3-R	CCAAGCAATGCCTTGAATACC			

AvPIP2;1-F	TACACCATCGGTGTTGGATTG	867	1.04	0.9917
AvPIP2;1-R	GAGTCTCTGGCACTTCTCTTTG			
AvPIP2;2-F	GGACTCCCATGTTCCTGTATTG	858	1.006	0.9908
AvPIP2;2-R	CAAAGCTTCGAGCAGGGTTA			
AvPIP2;3-F	TCTTGGATCTGCTGTCATCTTC	864	0.978	0.9956
AvPIP2;3-R	CAGCTCCTGCTCTCAAGATAAA			
AvPIP2;4-F	GCCTTGGGTGCTGAGATTAT	852	1.049	0.9981
AvPIP2;4-R	ATTGGAAGAGGAGCCAATACAG			
AvTIP1;1-F	GGACAAACCACTGGGTATACTG	759	1.017	0.9937
AvTIP1;1-R	AGTAGTCCGTGACTGGAATTTG			
AvTIP1;2-F	GATCAGCTGGACATGGACTAAC	756	1.018	0.9929
AvTIP1;2-R	GTGCTAGCGGTCAAGAAGATT			
AvTIP1;3-F	CACAGCCATTGACCCTAAGAA	783	1.039	0.9923
AvTIP1;3-R	AAACACCGCCCACAAGAA			
AvTIP2;1-F	ACTGGCATCTTCTACTGGATTG	747	1.026	0.9968
AvTIP2;1-R	CCAAAGAGTGGACCGGAATAG			
AvTIP2;2-F	CTAGCAGCAGGCCCATTTA	756	1.001	0.9982
AvTIP2;2-R	CCAACCCAGTAGATCCAATTCT			
AvTIP3;1-F	TGGGTTGGAGATGGCATAATC	774	1.047	0.9958
AvTIP3;1-R	CGGAGCATCAGCCTTTATGA			
AvTIP4;1-F	CTGCGTTGGTGGGTACATTA	747	0.988	0.9962
AvTIP4;1-R	TTGTCAGTCCACCGGTTAAG			
AvTIP5;1-F	CCTTTCACTGGTGGGTCAAT	756	0.951	0.9932
AvTIP5;1-R	CAATCAAGGGTCCTACCCAATAC			
AvActin-F	TTGTGAGGGATGTGAAAGAA	598	1.014	0.9925
AvActin-R	AATGAATGAAGGCTGGAAAA			

Table S2. ΔCT Values and ANOVA results from quantitative real-time PCR across different tissues and gene groups

	Root					Stem					Leaf				
Treatment	NC300	NHC300	NA300	4℃	40℃	NC300	NHC300	NA300	4℃	40℃	NC300	NHC300	NA300	4℃	40℃
NIP1;1	12.263ab	3.187cd	9.763b	11.597ab	-0.37cdef	13.64a	3.547c	-0.85ef	-3.153f	-0.227cdef	3.537c	-1.093ef	2.46cde	-2.223f	-0.463def
NIP1;2	0.307cd	5.55b	2.797bc	9.123a	-5.727f	4.63b	5.497b	-5.77f	-0.427cde	0.793cd	-0.05cde	-1.27de	-1.513de	-3.37ef	-0.253cde
NIP2;1	2.153de	7.703bc	4.747cd	12.327a	-3.283f	2.113de	9.927ab	-6.6g	-1.183ef	4.813cd	0.013ef	-1ef	-3.283f	-2.88f	1.67de
NIP3;1	1.6bc	4.713a	4.01ab	6.693a	-4.523de	4.143bc	5.273a	-7.023f	-1.8cd	0.593ab	0.683cd	-1.047cd	-1.893ef	-0.61de	1.687cd
NIP4;1	-1.11bc	9.063b	1.877b	13.417a	1.1ef	8.607bcd	6.003b	-4.077g	5.627ef	8.427b	1.21cde	3.433de	2.88fg	6.617ef	8.197bcd
NIP5;1	11.483b	4.357a	11.193b	9.933a	-1.953cd	4.177b	3.177a	-5.19d	-5.127cd	-2.867b	3.297cd	-2.207cd	0.67d	-2.197cd	-2.98bc
NIP6;1	6.657cd	3.327b	8.593c	9.593a	-1.713fg	1.697de	2.65ab	-6.087h	-8.317ef	-5.163c	-0.6e	-1.77e	-3.247gh	-2.373ef	-2.107e
NIP7;1	-2.257def	6.097bc	-0.667de	8.113a	-7.39g	0.327def	4.5ab	-8.11h	-3.133efg	2.353cd	-1.31efg	-2.11efg	-4.063h	-0.16fg	-0.863ef
PIP1;1	10.213b	3.41c	14.073a	12.033ab	-2.027d	13.67a	-2.12d	-1.857d	-8.36f	-5.703e	2.743c	-7.09ef	10.287b	-8.573f	3.637c
PIP1;2	10.857b	5.12c	13.153a	9.843b	-5.753hi	1.52e	-4.68gh	-6.43i	-10.373k	-8.83j	-4.173g	-9.81jk	-0.86f	-13.82l	3.637d
PIP1;3	0.99cd	-0.873d	1.727cd	4.973ab	-10.37f	7.26a	6.117ab	-5.203e	-4.77e	-4.32e	-0.97d	-4.653e	0.443cd	-8.743f	3.637bc
PIP2;1	10.58a	8.107b	10.95a	10.227a	-5.203h	5.517c	0.26d	-4.943gh	-2.783f	-3.007fg	-0.143de	0.623d	0.343d	-2.01ef	3.637c
PIP2;2	3.16cd	-2.2ef	3.653bcd	6.79a	-9.447i	5.46abc	2.117d	-3.543fg	-5.237gh	-3.883fg	-0.23e	-4.2fg	5.543ab	-6.633h	3.637bcd
PIP2;3	8.753b	3.217c	11.95a	12.24a	-2.13e	8.263b	-2.52e	-3.783e	-8.24fg	-6.127f	0.767d	-7.277f	5.28c	-10.157g	3.637c
PIP2;4	12.937ab	8.797bc	13.867ab	15.87a	-0.747de	4.88cd	-3.24ef	-5.343efg	-9.837gh	-8.283fgh	-0.64de	-9.253fgh	4.167cd	-13.287h	3.637cd
TIP1;1	1.13bcd	3.403bc	3.433bc	12.693a	-2.043def	4.327b	3.713bc	-7.243g	-3.043f	-0.807def	0.58cde	-0.667def	-0.723def	-2.583ef	-0.903def
TIP1;2	1.58cd	7.523b	4.293bc	11.863a	-3.2ef	4.03bc	6.387b	-5.807f	-1.22de	-1.113de	0.44de	-2.847ef	-2.397ef	-0.363de	-2.483ef
TIP1;3	-5.987de	-10.07f	-3.503cd	-1.54bc	-13.363g	5.817a	-1.397bc	-6.357de	-7.877ef	-5.973de	0.21b	-7.173ef	-1.097bc	-5.08de	-6.11de
TIP2;1	5.307bc	1.48cd	7.17b	13.397a	-8.783g	7.897b	-0.933de	-4.59ef	-6.587fg	-5.943fg	1.933cd	-6.227fg	1.96cd	-3.977ef	-7.287fg
TIP2;2	0.693def	3.693cd	2.017de	10.547a	-3.903gh	5.81bc	7.573ab	-5.52h	1.053def	1.99de	1.81def	0.34def	-0.463ef	-1.517fg	3.49cd
TIP3;1	-1.137cde	3.523b	0.873c	8.267a	-8.773i	-1.34cde	6.163a	-11.24j	-6.677hi	0.087cd	-3.87fg	-1.957def	-5.347gh	-2.367ef	-1.56def
TIP4;1	7.007ab	2.213cd	8.027ab	9.577a	-1.823ef	7.027ab	2.093cd	5.467bc	-6.89g	-3.163f	2.55cd	-3.347f	1.503de	-1.563ef	0.007def
TIP5;1	-3.84ef	5.287abc	-0.957de	6.397ab	-5.95f	5.203abc	9.017a	-3.453ef	1.33cd	7.06ab	-1.57def	2.59bcd	-2.127def	-0.29de	0.4de
SIP1;1	-7.42cdef	-7.707def	-3.137bcd	-4.847bcde	-15.033g	6.753a	-2.783bc	-9.593f	-8.167ef	-3.59bcde	-4.103bcde	-7.413cdef	-2.493b	-6.13bcdef	-5.123bcdef
XIP1;1	4.417ab	3.787ab	2.473bc	6.25a	-6.16ef	0.56cd	5.933a	-5.617ef	-3.933e	-5.397ef	-0.793d	-7.83f	0.91cd	-7.703f	3.637ab

Note: The table presents ΔCT values obtained from quantitative real-time PCR experiments, organized by treatment group, tissue type (root, stem, leaf), and gene family (NIP, PIP, TIP). The first row indicates the temperature or condition code, while subsequent rows list the specific ΔCT measurements for each sample. The data are followed by results of one-way analysis of variance (ANOVA) to assess significant differences in expression levels among different treatment groups. Values with different superscript letters indicate statistically significant differences at $p< 0.05$. This format allows for direct comparison of gene expression changes across experimental conditions. Abbreviations stand for the following: NC300, 300 mmol Na_2CO_3; NHC300, 300 mmol $NaHCO_3$; NA300, 300 mmol NaCl.

Table S3. Comparative physiological profiling of *A. venetum* under various stress conditions

	Plant height /cm	Root length /cm	Root fresh weight /g	Stem fresh weigh /g	Leaf fresh weigh /g	Root dry weight /mg	Stem dry weight /mg	Leaf dry weight /mg	Root water content /%	Stem water content /%	Leaf water content /%
CK	30.30±0.94	12.45±1.23	0.32±0.02	1.20±0.24	1.81±0.16	44.19±1.94	203.70±10.1	179.02±9.22	86.13±1.11	82.23±4.37	90.052±0.81
NaCl -100 mmol	40.45±2.54	21.94±0.89	0.39±0.05	1.56±0.06	0.85±0.08	79.14±3.75	237.64±9.43	192.36±9.7	79.48±1.79	84.75±0.74	87.65±0.76
NaCl -300 mmol	34.55±1.95	17.43±1.11	0.78±0.05	1.10±0.15	2.22±0.24	45.21±5.84	137.60±6.08	176.78±11.79	94.22±0.49	87.32±1.32	91.90±1.32
Na2CO3 -100 mmol	36.27±1.28	20.58±1.15	0.84±0.08	1.32±0.02	1.45±0.12	67.96±4.30	172.84±5.08	177.76±6.05	91.88±0.65	86.90±0.46	87.67±0.99
Na2CO3 -300 mmol	20.98±0.77	12.4±0.97	0.59±0.06	1.67±0.06	0.62±0.05	73.76±3.80	183.85±11.63	181.822±6.78	87.38±1.43	88.99±0.54	88.79±0.33
NaHCO3 -100 mmol	28.73±1.11	19.61±2.14	0.29±0.04	0.89±0.06	1.36±0.05	48.14±3.72	179.02±8.81	200.18±8.99	83.13±2.54	79.81±1.51	85.28±0.22
NaHCO3 -300 mmol	18.98±0.45	22.84±1.05	0.23±0.03	1.30±0.07	1.82±0.11	45.28±4.27	253.58±9.97	173.182±9.95	80.06±3.52	80.44±1.32	90.43±0.97
4℃	33.83±1.45	14.01±1.56	0.292±0.03	1.40±0.09	1.57±0.04	62.10±2.39	146.60±6.67	282.13±9.65	78.60±1.77	89.48±0.92	82.01±0.92
10℃	29.97±1.98	13.70±0.77	0.44±0.08	0.69±0.05	1.742±0.05	49.23±2.19	130.55±5.33	232.08±4.47	88.48±1.76	80.98±1.61	86.66±0.49
25℃	41.61±2.58	23.75±2.04	0.79±0.05	0.68±0.04	1.51±0.06	85.91±3.97	122.47±2.76	201.99±5.77	89.11±0.53	81.93±1.01	86.63±0.37
35℃	34.1±0.98	11.93±1.92	0.73±0.07	0.74±0.03	1.80±0.05	40.68±1.40	150.62±5.29	252.94±3.80	79.24±1.47	79.62±0.93	85.94±0.44
40℃	25.08±0.79	17.38±1.42	0.35±0.05	0.67±0.04	1.48±0.07	71.26±4.25	145.66±5.40	259.64±6.60	79.35±2.86	78.18±1.44	82.41±1.06

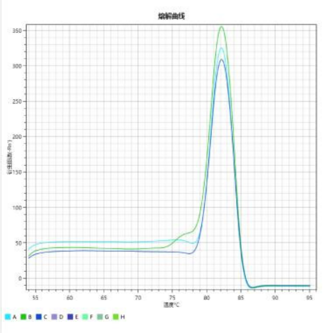

NIP1;1

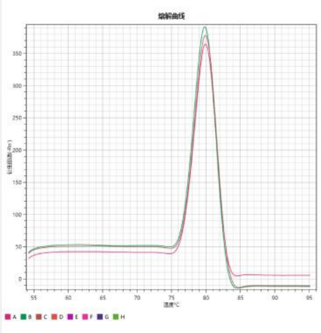

NIP1;2

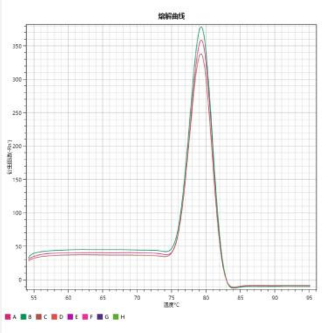

NIP2;1

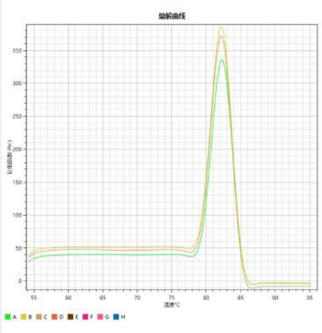

NIP3;1

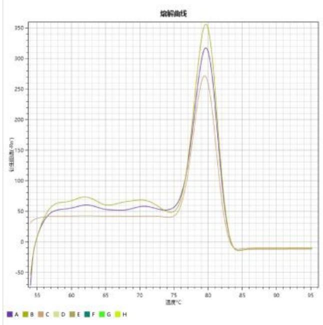

NIP4;1

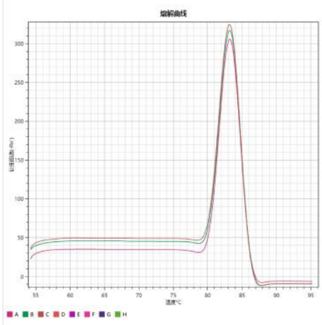

NIP5;1

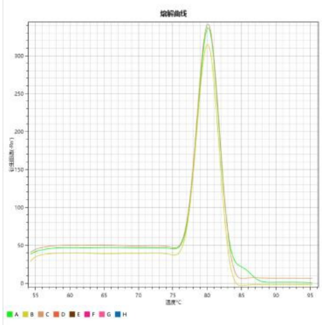

NIP6;1

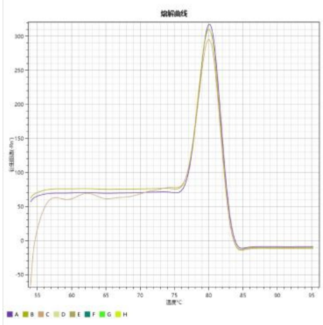

NIP7;1

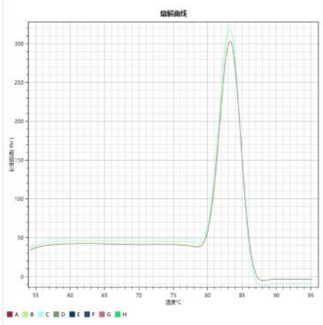

PIP1;1

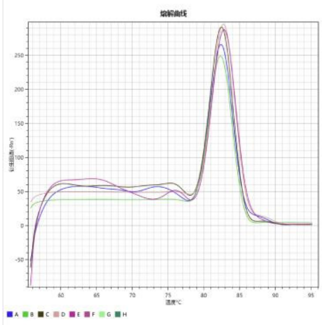

PIP1;2

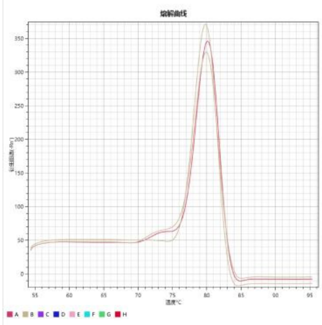

PIP1;3

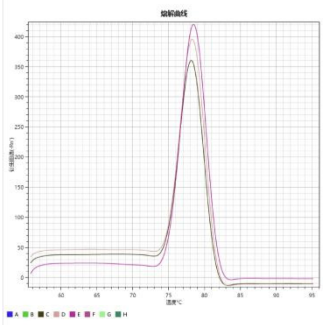

PIP2;1

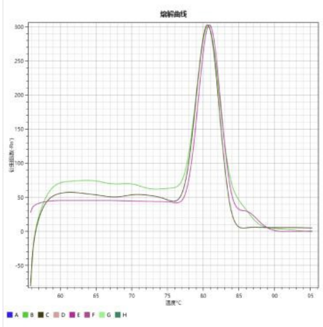

PIP2;2

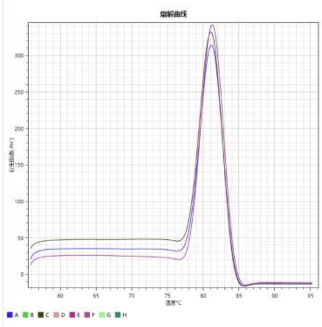

PIP2;3

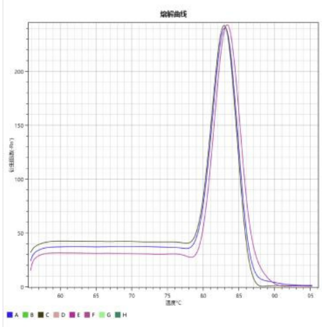

PIP2;4

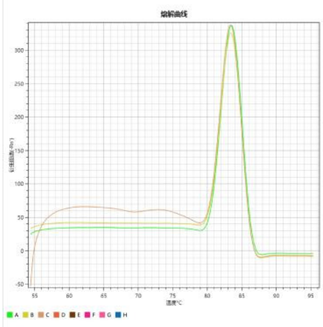

TIP1;1

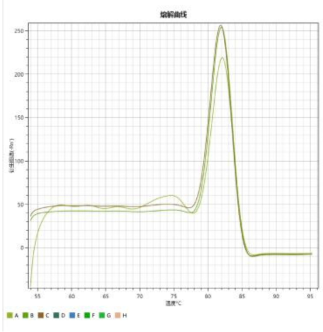

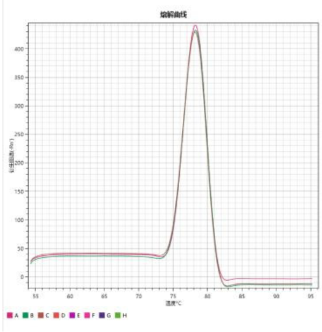

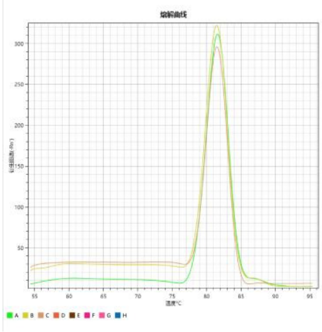

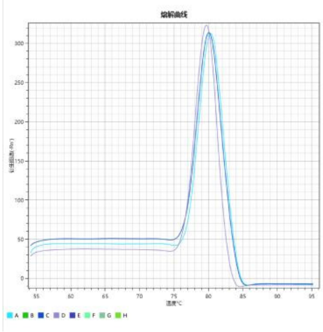

TIP1;2 TIP1;3 TIP2;1 TIP2;2

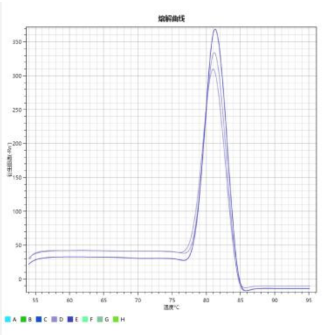

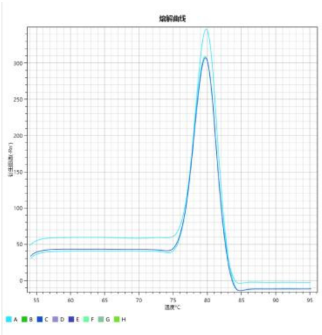

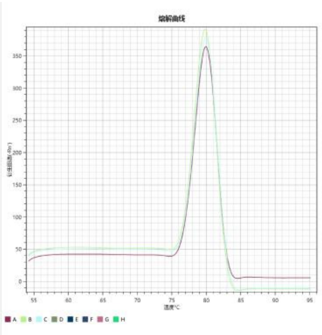

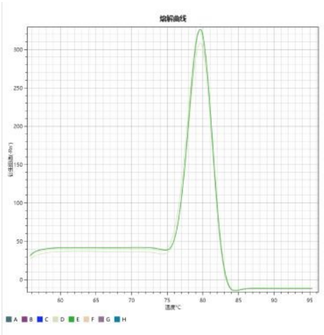

TIP3;1 TIP4;1 TIP5;1 SIP1;1

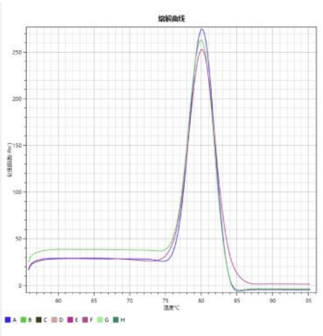

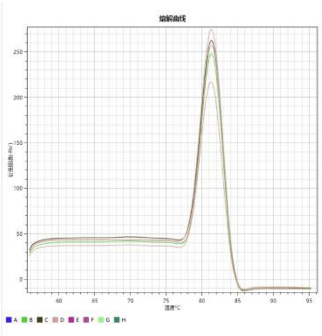

XIP1;1 ACTIN

Figure S1. Melting curve analysis of 25 aquaporin genes from *A. venetum*
